# Supplementary material for: Scientific progress made towards bridging the knowledge gap in the biology of Mediterranean marine fishes
Source: PLoS One. 2022 Nov 10;17(11):e0277383. doi: 10.1371/journal.pone.0277383 (PMC9648729; doi:10.1371/journal.pone.0277383)
Supplement: S1 Table — The commercial value (Val) is shown as price category (VH: very high; H: high; M: medium; L: low) and the protection status (IUCN) as IUCN Red List status category (LC: least concern; EN: endangered; DD: data deficient; NE: not evaluated; NT: near threatened; VU: vulnerable; CR: critically endangered). We included all the species with 8/8 and 7/8 studied characteristics and only the species with 6/8 studied characteristics and more than 40 records. (DOCX) [file pone.0277383.s001.docx]

**S1 Table. List of the most studied fish species in the Mediterranean Sea based on the number of records (Number of Rec.), the number of studied characteristics (Number of Char.) and the number of records per characteristic** (LWR: length-weight relationships; G: growth parameters; A: lifespan; Mat: length at maturity; Sp: onset and duration of spawning; Fec: fecundity; M: mortality; Diet: feeding preferences). The commercial value (Val) is shown as price category (VH: very high; H: high; M: medium; L: low) and the protection status (IUCN) as IUCN Red List status category (LC: least concern; EN: endangered; DD: data deficient; NE: not evaluated; NT: near threatened; VU: vulnerable; CR: critically endangered). We included all the species with 8/8 and 7/8 studied characteristics and only the species with 6/8 studied characteristics and more than 40 records.

| **Species** | **Common Name** | **Family** | **IUCN** | **Commercial Value** | **Number of Rec.** | **Number of Char.** | **Number of records per characteristic_**  **Present study** |
| --- | --- | --- | --- | --- | --- | --- | --- |
| *Merluccius merluccius* | European hake | Merlucciidae | LC | H | 227 | 8/8 | 59 LWR, 58 G, 29 Diet, 24Sp,23 Mat, 15 A, 10 Fec, 9 M |
| *Mullus barbatus barbatus* | Red mullet | Mullidae | LC | M | 163 | 8/8 | 49 LWR, 47 G, 19 Diet, 13 A, 12 Sp, 12 Mat, 7 Fec, 4 M |
| *Engraulis encrasicolus* | European anchovy | Engraulidae | LC | M | 142 | 8/8 | 39 LWR, 23 Sp, 23 G, 23 Diet, 13 A, 10 Mat, 7 Fec, 4 M |
| *Sardina pilchardus* | European pilchard | Clupeidae | LC | L | 125 | 8/8 | 29 G, 24 LWR, 22 Diet, 19 Sp, 11 Mat, 10 A, 5 Fec, 5 M |
| *Pagellus erythrinus* | Common pandora | Sparidae | LC | M | 124 | 8/8 | 49 LWR, 17 G,15 A, 14 Diet, 13 Sp, 12 Mat, 3 Fec, 1 M |
| *Mullus surmuletus* | Surmullet | Mullidae | LC | VH | 115 | 7/8 | 48 LWR, 18 Sp, 17 G, 14 Diet, 8 Mat, 7 A, 3 M, 0 Fec |
| *Diplodus annularis* | Annular seabream | Sparidae | LC | L | 115 | 8/8 | 57 LWR, 17 Sp, 13 G, 8 A, 7 Mat, 7 Diet, 4 Fec, 2 M |
| *Boops boops* | Bogue | Sparidae | LC | H | 100 | 8/8 | 36 LWR, 19 G, 15 Sp, 10 A, 9 Mat, 5 Diet, 3 M, 3 Fec |
| *Diplodus vulgaris* | Common two-banded seabream | Sparidae | LC | L | 80 | 8/8 | 30 LWR, 14 Diet, 10 Sp, 8 G, 7 Mat, 7 A, 3 M, 1 Fec |
| *Serranus cabrilla* | Comber | Serranidae | LC | M | 74 | 7/8 | 35 LWR, 9 Sp, 8 G, 8 Diet, 7 A, 4 Mat, 3 M, 0 Fec |
| *Thunnus thynnus* | Atlantic bluefin tuna | Scombridae | EN | VH | 73 | 8/8 | 16 Diet, 14LWR, 14 G, 10 Sp, 8 A, 5 Mat, 3 M, 3 Fec |
| *Trachurus trachurus* | Atlantic horse mackerel | Carangidae | VU | M | 70 | 8/8 | 25 LWR, 12 Diet, 10 Mat, 8 G, 6 Sp, 5 A, 3 M, 1 Fec |
| *Scorpaena porcus* | Black scorpionfish | Scorpaenidae | LC | L | 68 | 7/8 | 29 LWR, 15 Diet, 7 Sp, 6 G, 5 A, 5 Mat, 1 Fec, 0 M |
| *Pagellus acarne* | Axillary seabream | Sparidae | LC | M | 67 | 8/8 | 28 LWR, 9 Sp, 8 Mat, 7 G, 7 A, 4 Fec, 2 M, 2 Diet |
| *Lithognathus mormyrus* | Sand steenbras | Sparidae | LC | M | 66 | 7/8 | 27 LWR, 9 G, 8 Sp, 7 Mat, 7 Diet, 6 A, 2 M, 0 Fec |
| *Diplodus sargus* | White seabream | Sparidae | LC | VH | 64 | 8/8 | 24 LWR, 11 Diet, 10 Sp, 7 Mat, 4 G, 4 A, 3 Fec, 1 M |
| *Sardinella aurita* | Round sardinella | Clupeidae | LC | M | 63 | 7/8 | 20 LWR, 12 G, 10 Diet, 7 Sp, 6 Mat, 4 A, 4 Fec, 0 M |
| *Sparus aurata* | Gilthead seabream | Sparidae | LC | VH | 59 | 7/8 | 26 LWR, 11 G, 6 Sp, 5 A, 4 M, 4 Mat, 3 Diet, 0 Fec |
| *Trachurus mediterraneus* | Mediterranean horse mackerel | Carangidae | LC | L | 57 | 8/8 | 25 LWR, 12 Diet, 7 G, 4 A, 4 Sp, 2 M, 2 Mat, 1 Fec |
| *Spicara maena* | Blotched picarel | Sparidae | LC | NA | 56 | 7/8 | 28 LWR, 8 G, 5 Sp, 5 Diet, 4 A, 4 Mat, 2 Fec, 0 M |
| *Raja clavata* | Thornback ray | Rajidae | NT | M | 55 | 7/8 | 18 LWR, 12 Diet, 8 G, 6 A, 6 Mat, 3 Fec, 2 Sp, 0 M |
| *Sarpa salpa* | Salema | Sparidae | LC | M | 55 | 7/8 | 28 LWR, 12 Diet, 6 Sp, 4 Mat, 2 G, 2 A, 1 M, 0 Fec |
| *Galeus melastomus* | Blackmouth catshark | Pentanchidae | LC | H | 54 | 6/8 | 18 Diet, 15 Mat, 10 LWR, 8 Sp, 2 Fec, 1 G, 0 A, 0 M |
| *Chelidonichthys lucerna* | Tub gurnard | Triglidae | LC | M | 53 | 8/8 | 15 LWR, 9 Diet, 8 G, 6 Sp, 6 Mat, 5 A, 3 Fec, 1 M |
| *Spicara smaris* | Picarel | Sparidae | LC | M | 53 | 8/8 | 25 LWR, 10 G, 5 A, 4 Sp, 3 Diet, 2 M, 2 Mat, 2 Fec |
| *Chelon auratus* | Golden grey mullet | Mugilidae | LC | M | 51 | 8/8 | 22 LWR, 11 G, 5 A, 4 Sp, 3 M, 2 Mat, 2 Fec, 2 Diet |
| *Atherina boyeri* | Big-scale sand smelt | Atherinidae | LC | H | 50 | 8/8 | 21 LWR, 9 Sp, 7 G, 4 A, 4 Diet, 2 Mat, 2 Fec, 1 M |
| *Scyliorhinus canicula* | Lesser spotted dogfish | Scyliorhinidae | LC | M | 45 | 6/8 | 16 Diet, 12 LWR, 8 Mat, 6 Fec, 2 Sp, 1 G, 0 A, 0 M |
| *Chelon ramada* | Thinlip grey mullet | Mugilidae | LC | M | 44 | 8/8 | 16 LWR, 8 Mat, 5 G, 5 Sp, 5 Fec, 2 A, 2 Diet, 1 M |
| *Micromesistius poutassou* | Blue whiting | Gadidae | NA | L | 44 | 8/8 | 12 LWR, 11 G, 8 Diet, 5 Sp, 3 A, 3 Mat, 1 M, 1 Fec |
| *Serranus hepatus* | Brown comber | Serranidae | LC | M | 44 | 6/8 | 26 LWR, 7 Diet, 5 G, 3 Sp, 2 Mat, 1 A, 0 M, 0 Fec |
| *Solea solea* | Common sole | Soleidae | DD | VH | 44 | 7/8 | 18 LWR, 9 G, 7 Sp, 5 Diet, 3 Mat, 1 A, 1 Fec, 0 M |
| *Dicentrarchus labrax* | European seabass | Moronidae | LC | VH | 43 | 8/8 | 19 LWR, 8 Sp, 6 G, 3 Mat, 3 Fec, 2 A, 1 M, 1 Diet |
| *Uranoscopus scaber* | Stargazer | Uranoscopidae | LC | NA | 42 | 8/8 | 20 LWR, 6 Diet, 4 G, 4 Mat, 3 Sp, 2 A, 2 Fec, 1 M |
| *Serranus scriba* | Painted comber | Serranidae | LC | M | 41 | 6/8 | 22 LWR, 7 Sp, 5 Diet, 3 G, 3 Mat, 1 A, 0 M, 0 Fec |
| *Spondyliosoma cantharus* | Black seabream | Sparidae | LC | VH | 41 | 7/8 | 15 LWR, 6 G, 5 A, 5 Sp, 4 Mat, 4 Diet, 2 Fec, 0 M |
| *Dentex dentex* | Common dentex | Sparidae | VU | VH | 39 | 7/8 | 15 LWR, 6 Sp, 5 Mat, 4 G, 4 A, 3 Diet, 2 Fec, 0 M |
| *Xiphias gladius* | Swordfish | Xiphiidae | LC | VH | 39 | 7/8 | 10 G, 8 Diet, 7 Sp, 5 A, 4 LWR, 4 Mat, 1 Fec, 0 M |
| *Scorpaena notata* | Small red scorpionfish | Scorpaenidae | LC | L | 38 | 7/8 | 19 LWR, 8 Diet, 3 G, 3 Sp, 2 A, 2 Mat, 1 Fec, 0 M |
| *Helicolenus dactylopterus* | Blackbelly rosefish | Sebastidae | LC | L | 37 | 7/8 | 10 LWR, 7 Sp, 6 G, 6 Diet, 3 A, 3 Fec, 2 Mat, 0 M |
| *Squalus blainville* | Longnose spurdog | Squalidae | DD | M | 37 | 7/8 | 9 LWR, 9 Diet, 7 Sp, 5 G, 3 Sp, 2 A, 2 Fec, 0 M |
| *Symphodus tinca* | East Atlantic peacock wrasse | Labridae | LC | VH | 37 | 7/8 | 16 LWR, 7 Diet, 5 G, 4 Sp, 3 Mat, 1 A, 1 Fec, 0 M |
| *Lepidotrigla cavillone* | Large-scaled gurnard | Triglidae | NA | L | 36 | 8/8 | 12 LWR, 6 Diet, 5 Sp, 4 G, 4 A, 3 Mat, 1 M, 1 Fec |
| *Belone belone* | Garfish | Belonidae | LC | H | 35 | 8/8 | 14 LWR, 5 Sp, 5 Diet, 4 G, 2 A, 2 Mat, 2 Fec, 1 M |
| *Chelon saliens* | Leaping mullet | Mugilidae | LC | M | 35 | 8/8 | 13 LWR, 7 G, 5 Sp, 3 Mat, 2 A, 2 M, 2 Fec, 1 Diet |
| *Epinephelus marginatus* | Dusky grouper | Serranidae | VU | VH | 35 | 8/8 | 8 LWR, 8 Diet, 6 Sp, 4 A, 4 Mat, 3 G, 1 M, 1 fec |
| *Oblada melanura* | Saddled seabream | Sparidae | LC | VH | 34 | 8/8 | 10 LWR, 6 Diet, 5 Sp, 4 G, 3 A,3 M, 2 Mat, 1 Fec |
| *Sciaena umbra* | Brown meagre | Sciaenidae | NT | VH | 34 | 8/8 | 11 LWR, 7 Sp, 4 G, 4 Diet, 3 Mat, 2 A, 2 M, 1 Fec |
| *Chelon labrosus* | Thicklip grey mullet | Mugilidae | LC | L | 33 | 8/8 | 16 LWR, 5 G, 4 Sp, 3 Mat, 2 M, 1 A, 1 Fec, 1 Diet |
| *Dipturus oxyrinchus* | Longnosed skate | Rajidae | NT | M | 32 | 8/8 | 8 LWR, 7 Diet, 5 Mat, 4 G, 3 Sp, 2 A, 2 Fec, 1 M |
| *Mugil cephalus* | Flathead grey mullet | Mugilidae | LC | VH | 32 | 8/8 | 14 LWR, 7 G, 5 Sp, 2 Mat, 1 A, 1 M, 1 fec, 1 Diet |
| *Scomber colias* | Atlantic chub mackerel | Scombridae | LC | VH | 32 | 8/8 | 13 LWR, 5 G, 4 Sp, 3 A, 3 Diet, 2 M, 1 Mat. 1 Fec |
| *Upeneus moluccensis* | Goldband goatfish | Mullidae | LC | H | 30 | 8/8 | 12 LWR, 5 Diet, 4 Mat, 3 G, 2 A, 2 Fec, 1 M, 1 Sp |
| *Zosterisessor ophiocephalus* | Grass goby | Gobiidae | LC | VH | 28 | 8/8 | 10 LWR, 4 G, 4 Sp, 3 A, 2 M, 2 Mat, 2 Fec, 1 Diet |
| *Raja miraletus* | Brown ray | Rajidae | LC | M | 26 | 8/8 | 8 LWR, 6 Diet, 3 G, 3 Sp, 3 Mat, 1 A, 1 M, 1 Fec |
| *Upeneus pori* | Por's goatfish | Mullidae | LC | NA | 26 | 8/8 | 9 LWR, 4 G, 3 A, 3 Mat, 3 Diet, 2 Sp, 1 M, 1 Fec |
| *Lophius piscatorius* | Angler | Lophiidae | LC | H | 21 | 8/8 | 9 LWR, 4 Diet, 2 Sp, 2 Mat, 1 G, 1 A, 1 M, 1 Fec |
| *Sargocentron rubrum* | Redcoat | Holocentridae | LC | M | 18 | 8/8 | 5 LWR, 3 Sp, 2 G, 2 A, 2 M, 2 Diet, 1 Mat, 1 Fec |
| *Aphia minuta* | Transparent goby | Gobiidae | NA | VH | 15 | 8/8 | 4 Sp, 3 LWR, 2 A, 2 Diet, 1 G, 1 M, 1 Mat, 1 Fec |
