# Supplementary material for: Scientific progress made towards bridging the knowledge gap in the biology of Mediterranean marine fishes
Source: PLoS One. 2022 Nov 10;17(11):e0277383. doi: 10.1371/journal.pone.0277383 (PMC9648729; doi:10.1371/journal.pone.0277383)
Supplement: S2 Table — (LWR: length-weight relationships; G: growth parameters; A: lifespan; Mat: length at maturity; Sp: onset and duration of spawning; Fec: fecundity; M: mortality; Diet: feeding preferences). The commercial value (Val) is shown as price category (VH: very high; H: high; M: medium; L: low) and the protection status (IUCN) as IUCN Red List status category (LC: least concern; EN: endangered; DD: data deficient; NE: not evaluated; NT: near threatened; VU: vulnerable; CR: critically endangered). (DOCX) [file pone.0277383.s002.docx]

**S2 Table. List of all the Mediterranean chondrichthyan species with or without studies on their biological characteristics.** (LWR: length-weight relationships; G: growth parameters; A: lifespan; Mat: length at maturity; Sp: onset and duration of spawning; Fec: fecundity; M: mortality; Diet: feeding preferences). The commercial value (Val) is shown as price category (VH: very high; H: high; M: medium; L: low) and the protection status (IUCN) as IUCN Red List status category (LC: least concern; EN: endangered; DD: data deficient; NE: not evaluated; NT: near threatened; VU: vulnerable; CR: critically endangered).

| **Species** | **Common Name** | **Family** | **IUCN** | **Commercial Value** | **Number of Rec.** | **Number of Char.** | **Number of records per characteristic** |
| --- | --- | --- | --- | --- | --- | --- | --- |
| *Raja clavata* | Thornback ray | Rajidae | NT | M | 55 | 7 | 18 LWR, 12 Diet, 8 G, 6 A, 6 Mat, 3 Fec, 2 Sp |
| *Galeus melastomus* | Blackmouth catshark | Pentanchidae | LC | H | 54 | 6 | 18 Diet, 15 Mat, 10 LWR, 8 Sp, 2 Fec, 1 G |
| *Scyliorhinus canicula* | Lesser spotted dogfish | Scyliorhinidae | LC | M | 45 | 6 | 16 Diet, 12 LWR, 8 Mat, 6 Fec, 2 Sp, 1 G |
| *Squalus blainville* | Longnose spurdog | Squalidae | DD | M | 37 | 7 | 9 LWR, 9 Diet, 7 Mat, 5 G, 3 Sp, 2 A, 2 Fec |
| *Torpedo marmorata* | Marbled electric ray | Torpedinidae | DD | NA | 34 | 7 | 14 LWR, 6 Diet, 5 Mat, 4 Fec, 3 Sp, 1 G, 1 A |
| *Dipturus oxyrinchus* | Longnosed skate | Rajidae | NT | M | 32 | 8 | 8 LWR, 7 Diet, 5 Mat, 4 G, 3 Sp, 2 A, 2 Fec, 1 M |
| *Etmopterus spinax* | Velvet belly | Etmopteridae | LC | NA | 26 | 5 | 10 Diet, 6 LWR, 5 Mat, 4 Sp, 1 Fec |
| *Raja miraletus* | Brown ray | Rajidae | LC | M | 26 | 8 | 8 LWR, 6 Diet, 3 G, 3 Sp, 3 Mat, 1 A, 1 M, 1 Fec |
| *Squalus acanthias* | Picked dogfish | Squalidae | VU | M | 24 | 7 | 6 Diet, 5 LWR, 5 Mat, 2 G, 2 A, 2 Sp, 2 Fec |
| *Rhinobatos rhinobatos* | Common guitarfish | Rhinobatidae | EN | L | 21 | 7 | 5 Mat, 4 LWR, 4 Diet, 3 Sp, 3 Fec, 1 G, 1 A |
| *Torpedo torpedo* | Common torpedo | Torpedinidae | DD | NA | 20 | 7 | 6 Diet, 4 LWR, 3 Mat, 3 Fec, 2 G, 1 A, 1 Sp |
| *Mustelus mustelus* | Smooth hound | Triakidae | VU | M | 18 | 7 | 6 Diet, 5 LWR, 3 Mat, 1 G. 1 A, 1 Sp, 1 Fec |
| *Squatina aculeata* | Sawback angelshark | Squatinidae | CR | M | 16 | 5 | 6 LWR, 3 Sp, 3 Mat, 3 Fec, 1 Diet |
| *Raja asterias* | Mediterranean starry ray | Rajidae | NT | M | 15 | 4 | 7 Diet, 5 LWR, 2 Mat, 1 Fec |
| *Raja radula* | Rough ray | Rajidae | EN | M | 15 | 7 | 7 LWR, 3 Diet, 1 G, 1 A, 1 Sp, 1 Mat, 1 Fec |
| *Tetronarce nobiliana* | Electric ray | Torpedinidae | DD | NA | 15 | 6 | 8 LWR, 2 A, 2 Diet, 1 G, 1 Sp, 1 Fec |
| *Raja polystigma* | Speckled ray | Rajidae | LC | NA | 13 | 7 | 4 Diet, 3 LWR, 2 Fec, 1 G, 1 A, 1 Sp, 1 Mat |
| *Mustelus punctulatus* | Blackspotted smooth hound | Triakidae | DD | M | 12 | 5 | 5 Diet, 2 LWR, 2 Sp, 2 Mat, 1 Fec |
| *Chimaera monstrosa* | Rabbit fish | Chimaeridae | VU | L | 12 | 4 | 4 LWR, 4 Diet, 3 Sp, 1 Mat |
| *Dalatias licha* | Kitefin shark | Dalatiidae | VU | M | 11 | 4 | 6 Diet, 3 LWR, 1 Sp, 1 Mat |
| *Hexanchus griseus* | Bluntnose sixgill shark | Hexanchidae | NT | NA | 11 | 5 | 4 LWR, 4 Diet, 1 Sp, 1 Mat, 1 Fec |
| *Myliobatis aquila* | Common eagle ray | Myliobatidae | DD | H | 11 | 4 | 5 LWR, 3 Diet, 2 Sp, 1 Fec |
| *Aetomylaeus bovinus* | Bull ray | Myliobatidae | CR | M | 11 | 6 | 4 LWR, 2 Sp, 2 Diet, 1 G, 1 A, 1 Fec |
| *Oxynotus centrina* | Angular roughshark | Oxynotidae | VU | H | 11 | 5 | 5 LWR, 2 Sp, 2 Diet, 1 Mat, 1 Fec |
| *Raja brachyura* | Blonde ray | Rajidae | NT | M | 10 | 7 | 3 Diet, 2 LWR, 1 G, 1 A, 1 Sp, 1 Mat, 1 Fec |
| *Gymnura altavela* | Spiny butterfly ray | Gymnuridae | VU | H | 10 | 7 | 3 LWR, 2 Diet, 1 G, 1 A, 1 M, 1 Mat, 1 Fec |
| *Dasyatis marmorata* | Marbled stingray | Dasyatidae | DD | NA | 10 | 6 | 4 LWR, 2 Mat, 1 G, 1 A, 1 Fec, 1 Diet |
| *Centroscymnus coelolepis* | Portuguese dogfish | Somniosidae | NT | L | 8 | 4 | 5 Diet, 1 LWR, 1 Sp, 1 Mat |
| *Heptranchias perlo* | Sharpnose sevengill shark | Hexanchidae | NT | NA | 8 | 5 | 4 LWR, 1 Sp, 1 Mat, 1 Fec, 1 Diet |
| *Alopias superciliosus* | Bigeye thresher | Alopiidae | VU | L | 8 | 1 | 8 LWR |
| *Rostroraja alba* | White skate | Rajidae | EN | M | 8 | 2 | 7 LWR, 1 Fec |
| *Leucoraja circularis* | Sandy ray | Rajidae | EN | M | 7 | 4 | 4 LWR, 1 A, 1 Sp, 1 Diet |
| *Scyliorhinus stellaris* | Nursehound | Scyliorhinidae | ΝΤ | Μ | 6 | 5 | 2 LWR, 1 Sp, 1 Mat, 1 Fec, 1 Diet |
| *Carcharhinus plumbeus* | Sandbar shark | Carcharhinidae | VU | M | 6 | 3 | 3 Sp, 2 LWR, 1 Mat |
| *Prionace glauca* | Blue shark | Carcharhinidae | NT | M | 6 | 4 | 2 G, 2 A, 1 LWR, 1 Sp |
| *Squatina squatina* | Angelshark | Squatinidae | CR | M | 6 | 4 | 3 LWR, 1 Sp, 1 Mat, 1 Fec |
| *Centrophorus granulosus* | Gulper shark | Centrophoridae | EN | H | 5 | 3 | 3 Diet, 1 LWR, 1 Sp |
| *Carcharodon carcharias* | Great white shark | Lamnidae | VU | L | 5 | 2 | 3 Diet, 2 LWR, 1 G, 1 A, 1 Sp, 1 Mat, 1 Fec |
| *Centrophorus uyato* | Little gulper shark | Centrophoridae | EN | NA | 5 | 4 | 2 LWR, 1 Sp, 1 MAt, 1 Diet |
| *Squalus megalops* | Shortnose spurdog | Squalidae | LC | M | 5 | 5 | 1 LWR, 1 G, 1 A, 1 Mat, 1 Diet |
| *Squatina oculata* | Smoothback angelshark | Squatinidae | CR | M | 5 | 4 | 2 LWR, 1 Sp, 1 Fec, 1 Diet |
| *Carcharhinus limbatus* | Blacktip shark | Carcharhinidae | NT | M | 5 | 2 | 4 Mat, 1 Sp |
| *Leucoraja melitensis* | Maltese ray | Rajidae | CR | NA | 5 | 3 | 3 LWR, 1 Sp, 1 Fec |
| *Pteroplatytrygon violacea* | Pelagic stingray | Dasyatidae | LC | NA | 4 | 2 | 3 Diet, 1 Fec |
| *Leucoraja naevus* | Cuckoo ray | Rajidae | LC | M | 4 | 2 | 2 LWR, 2 Diet |
| *Galeorhinus galeus* | Tope shark | Triakidae | CR | M | 4 | 4 | 1 G, 1 Mat, 1 Fec, 1 Diet |
| *Raja montagui* | Spotted ray | Rajidae | LC | M | 3 | 2 | 2 LWR, 1 Diet |
| *Somniosus rostratus* | Little sleeper shark | Somniosidae | LC | NA | 3 | 2 | 2 LWR, 1 Diet |
| *Dasyatis tortonesei* | Tortonese's stingray | Dasyatidae | NA | NA | 3 | 3 | 1 LWR, 1 Mat, 1 Fec |
| *Lamna nasus* | Porbeagle | Lamnidae | VU | M | 3 | 1 | 3 LWR |
| *Mustelus asterias* | Starry smooth hound | Triakidae | LC | M | 3 | 3 | 1 LWR, 1 Mat, 1 Fec |
| *Alopias vulpinus* | Thresher | Alopiidae | VU | H | 2 | 2 | 1 LWR, 1 Diet |
| *Galeocerdo cuvier* | Tiger shark | Carcharhinidae | NT | M | 2 | 2 | 1 LWR, 1 Diet |
| *Carcharhinus altimus* | Bignose shark | Carcharhinidae | NT | M | 2 | 2 | 1 LWR, 1 Sp |
| *Carcharhinus brevipinna* | Spinner shark | Carcharhinidae | VU | M | 2 | 2 | 1 LWR, 1 Mat |
| *Dasyatis chrysonota* | Blue stingray | Dasyatidae | NT | NA | 2 | 2 | 1 LWR, 1 Sp |
| *Echinorhinus brucus* | Bramble shark | Echinorhinidae | DD | NA | 2 | 1 | 2 LWR |
| *Rhinoptera marginata* | Lusitanian cownose ray | Rhinopteridae | NT | M | 2 | 1 | 2 LWR |
| *Carcharhinus brachyurus* | Copper shark | Carcharhinidae | VU | H | 1 | 1 | 1 LWR |
| *Carcharhinus falciformis* | Silky shark | Carcharhinidae | VU | H | 1 | 1 | 1 LWR |
| *Carcharias taurus* | Sand tiger shark | Carchariidae | VU | NA | 1 | 1 | 1 LWR |
| *Cetorhinus maximus* | Basking shark | Cetorhinidae | EN | L | 1 | 1 | 1 LWR |
| *Himantura uarnak* | Honeycomb stingray | Dasyatidae | VU | L | 1 | 1 | 1 LWR |
| *Taeniura grabata* | Round stingray | Dasyatidae | DD | L | 1 | 1 | 1 LWR |
| *Glaucostegus halavi* | Halavi ray | Glaucostegidae | CR | NA | 1 | 1 | 1 LWR |
| *Hexanchus nakamurai* | Bigeyed sixgill shark | Hexanchidae | DD | NA | 1 | 1 | 1 LWR |
| *Isurus oxyrinchus* | Shortfin mako | Lamnidae | NT | M | 1 | 1 | 1 LWR |
| *Leucoraja fullonica* | Shagreen ray | Rajidae | VU | L | 1 | 1 | 1 LWR |
| *Raja undulata* | Undulate ray | Rajidae | EN | M | 1 | 1 | 1 LWR |
| *Carcharhinus amboinensis* | Pigeye shark | Carcharhinidae | DD | M | 0 | 0 |  |
| *Carcharhinus longimanus* | Oceanic whitetip shark | Carcharhinidae | CR | M | 0 | 0 |  |
| *Carcharhinus obscurus* | Dusky shark | Carcharhinidae | EN | M | 0 | 0 |  |
| *Galeus atlanticus* | Atlantic sawtail cat shark | Pentanchidae | NT | NA | 0 | 0 |  |
| *Pristis pectinata* | Smalltooth sawfish | Pristidae | CR | M | 0 | 0 |  |
| *Pristis pristis* | Common sawfish | Pristidae | CR | M | 0 | 0 |  |
| *Dipturus batis* | Blue skate | Rajidae | CR | M | 0 | 0 |  |
| *Raja africana* | African ray | Rajidae | NA | NA | 0 | 0 |  |
| *Raja rondeleti* | Rondelet's ray | Rajidae | NA | NA | 0 | 0 |  |
| *Sphyrna lewini* | Scalloped hammerhead | Sphyrnidae | CR | M | 0 | 0 |  |
| *Sphyrna mokarran* | Great hammerhead | Sphyrnidae | CR | H | 0 | 0 |  |
| *Sphyrna tudes* | Smalleye hammerhead | Sphyrnidae | CR | H | 0 | 0 |  |
| *Sphyrna zygaena* | Smooth hammerhead | Sphyrnidae | VU | L | 0 | 0 |  |
