# Supplementary material for: Scientific progress made towards bridging the knowledge gap in the biology of Mediterranean marine fishes
Source: PLoS One. 2022 Nov 10;17(11):e0277383. doi: 10.1371/journal.pone.0277383 (PMC9648729; doi:10.1371/journal.pone.0277383)
Supplement: S3 Table — The commercial value (Val) is shown as price category (VH: very high; H: high; M: medium; L: low) and the protection status (IUCN) as IUCN Red List status category (LC: least concern; EN: endangered; DD: data deficient; NE: not evaluated; NT: near threatened; VU: vulnerable; CR: critically endangered). For all the species of this table, commercial value is not known (“NA”) and both the number of records and characteristics are equal to 0. (DOCX) [file pone.0277383.s003.docx]

**S3 Table. List of some of the least studied families of non-commercial fish species in the Mediterranean Sea based on the number of studied characteristics and the number of records per characteristic**. The commercial value (Val) is shown as price category (VH: very high; H: high; M: medium; L: low) and the protection status (IUCN) as IUCN Red List status category (LC: least concern; EN: endangered; DD: data deficient; NE: not evaluated; NT: near threatened; VU: vulnerable; CR: critically endangered). For all the species of this table, commercial value is not known (“NA”) and both the number of records and characteristics are equal to 0.

| **Species** | **Common Name** | **Family** | **IUCN** |
| --- | --- | --- | --- |
| *Lipophrys pholis* | Shanny | Blenniidae | LC |
| *Omobranchus punctatus* | Muzzled blenny | Blenniidae | LC |
| *Parablennius pilicornis* | Ringneck blenny | Blenniidae | LC |
| *Parablennius thysanius* | Tasseled blenny | Blenniidae | LC |
| *Petroscirtes ancylodon* | Arabian fangblenny | Blenniidae | LC |
| *Salaria basilisca* |  | Blenniidae | LC |
| *Scartella cristata* | Molly miller | Blenniidae | LC |
| *Apletodon incognitus* |  | Gobiesocidae | LC |
| *Diplecogaster bimaculata* | Two-spotted clingfish | Gobiesocidae | LC |
| *Diplecogaster umutturali* |  | Gobiesocidae | NA |
| *Gouania willdenowi* | Blunt-snouted clingfish | Gobiesocidae | LC |
| *Lepadogaster candolii* | Connemarra clingfish | Gobiesocidae | NA |
| *Lepadogaster purpurea* | Cornish sucker | Gobiesocidae | LC |
| *Opeatogenys gracilis* |  | Gobiesocidae | LC |
| *Bathygobius soporator* | Frillfin goby | Gobiidae | LC |
| *Benthophilus stellatus* | Stellate tadpole-goby | Gobiidae | LC |
| *Buenia lombartei* |  | Gobiidae | NA |
| *Chromogobius quadrivittatus* | Chestnut goby | Gobiidae | LC |
| *Chromogobius zebratus* | Kolombatovic's goby | Gobiidae | LC |
| *Corcyrogobius liechtensteini* | Liechtenstein's goby | Gobiidae | LC |
| *Coryogalops ocheticus* |  | Gobiidae | EN |
| *Deltentosteus collonianus* | Toothed goby | Gobiidae | LC |
| *Didogobius bentuvii* | Ben-Tuvia's goby | Gobiidae | DD |
| *Didogobius schlieweni* |  | Gobiidae | LC |
| *Didogobius splechtnai* |  | Gobiidae | LC |
| *Favonigobius melanobranchus* | Blackthroat goby | Gobiidae | NT |
| *Gammogobius steinitzi* | Steinitz's goby | Gobiidae | LC |
| *Gobius couchi* | Couch's goby | Gobiidae | LC |
| *Gobius gasteveni* | Steven's goby | Gobiidae | LC |
| *Gobius kolombatovici* |  | Gobiidae | NA |
| *Gobius strictus* | Schmidt's goby | Gobiidae | DD |
| *Gobius xanthocephalus* |  | Gobiidae | LC |
| *Gymnesigobius medits* |  | Gobiidae | NA |
| *Lebetus guilleti* | Guillet's goby | Gobiidae | LC |
| *Lebetus patzneri* |  | Gobiidae | NA |
| *Lesueurigobius sanzi* | Sanzo's goby | Gobiidae | LC |
| *Millerigobius macrocephalus* |  | Gobiidae | LC |
| *Odondebuenia balearica* | Coralline goby | Gobiidae | LC |
| *Pomatoschistus knerii* | Kner's goby | Gobiidae | LC |
| *Pomatoschistus tortonesei* | Tortonese's goby | Gobiidae | EN |
| *Speleogobius trigloides* | Grotto goby | Gobiidae | LC |
| *Thorogobius macrolepis* | Large-scaled goby | Gobiidae | LC |
| *Tridentiger trigonocephalus* | Chameleon goby | Gobiidae | LC |
| *Trypauchen vagina* |  | Gobiidae | LC |
| *Vanderhorstia mertensi* | Mertens' prawn-goby | Gobiidae | LC |
| *Vanneaugobius dollfusi* |  | Gobiidae | NA |
| *Vanneaugobius pruvoti* |  | Gobiidae | LC |
| *Eretmophorus kleinenbergi* |  | Moridae | LC |
| *Guttigadus latifrons* |  | Moridae | NA |
| *Lepidion guentheri* |  | Moridae | LC |
| *Rhynchogadus hepaticus* |  | Moridae | DD |
| *Diogenichthys atlanticus* | Longfin lanternfish | Myctophidae | LC |
| *Gonichthys cocco* | Cocco’s lanternfish | Myctophidae | LC |
| *Lobianchia gemellarii* | Cocco's lantern fish | Myctophidae | LC |
| *Notoscopelus bolini* |  | Myctophidae | LC |
| *Notoscopelus kroyeri* | Lancet fish | Myctophidae | LC |
| *Symbolophorus veranyi* | Large-scale lantern fish | Myctophidae | LC |
| *Entelurus aequoreus* | Snake pipefish | Syngnathidae | NA |
| *Hippocampus fuscus* | Sea pony | Syngnathidae | NA |
| *Minyichthys sentus* |  | Syngnathidae | LC |
| *Nerophis lumbriciformis* | Worm pipefish | Syngnathidae | LC |
| *Syngnathus rostellatus* | Nilsson's pipefish | Syngnathidae | LC |
| *Syngnathus tenuirostris* | Narrow-snouted pipefish | Syngnathidae | DD |
