# Supplementary material for: Scientific progress made towards bridging the knowledge gap in the biology of Mediterranean marine fishes
Source: PLoS One. 2022 Nov 10;17(11):e0277383. doi: 10.1371/journal.pone.0277383 (PMC9648729; doi:10.1371/journal.pone.0277383)
Supplement: S4 Table — The commercial value (Val) is shown as price category (VH: very high; H: high; M: medium; L: low) and the protection status (IUCN) as IUCN Red List status category (LC: least concern; EN: endangered; DD: data deficient; NE: not evaluated; NT: near threatened; VU: vulnerable; CR: critically endangered). The differences between the results of the present study and the Dimarchopoulou et al. (2017) [1] study are highlighted in bold. (DOCX) [file pone.0277383.s004.docx]

**S4 Table.** **Comparison between current and previously published records on the biology of selected Mediterranean fish species (Dimarchopoulou et al., 2017), based on the number of studied characteristics and the number of records per characteristic** (LWR: length-weight relationships; G: growth parameters; A: lifespan; Mat: length at maturity; Sp: onset and duration of spawning; Fec: fecundity; M: mortality; Diet: feeding preferences). The commercial value (Val) is shown as price category (VH: very high; H: high; M: medium; L: low) and the protection status (IUCN) as IUCN Red List status category (LC: least concern; EN: endangered; DD: data deficient; NE: not evaluated; NT: near threatened; VU: vulnerable; CR: critically endangered). The differences between the results of the present study and the Dimarchopoulou et al. (2017) study are highlighted in bold.

| **Species** | **Common Name** | **Family** | **IUCN** | **Commercial Value** | **Number of Rec._**  **Present study** | **Number of Rec._ Dimarchopoulou *et al.* 2017** | **Number of Char._**  **Present study** | **Number of Char._ Dimarchopoulou *et al.* 2017** | **Number of records per characteristic_ Present study** | **Number of records per characteristic_ Dimarchopoulou**  ***et al.* 2017** |
| --- | --- | --- | --- | --- | --- | --- | --- | --- | --- | --- |
| **Species with commercial value** | | | | | | | | | | |
| *Arnoglossus thori* | Thor's scaldfish | Bothidae | DD | VH | **10** | 9 | 3/8 | 3/8 | **7 LWR**, 2 Diet, 1 Sp | 6 LWR, 2 Diet, 1 Sp |
| *Platichthys flesus* | European flounder | Pleuronectidae | LC | VH | **10** | 8 | 3/8 | 3/8 | **6 LWR**, 2 G, 2 Sp | 4 LWR, 2 G, 2 Sp |
| *Chelidonichthys obscurus* | Longfin gurnard | Triglidae | LC | VH | **8** | 7 | 3/8 | 3/8 | 4 Diet, **3 LWR**, 1 Sp | 4 Diet, 2 LWR, 1 Sp |
| *Epinephelus costae* | Goldblotch grouper | Serranidae | DD | VH | **10** | 7 | 3/8 | 3/8 | **6 LWR**, 2 G, **2 Diet** | 4 LWR, 2 G, 1 Diet |
| *Gobius cobitis* | Giant goby | Gobiidae | NA | VH | **9** | 7 | 3/8 | 3/8 | 4 LWR, 3 A, 2 Sp | 3 A, 2 LWR, 2 Sp |
| *Microchirus variegatus* | Thickback sole | Soleidae | LC | VH | 6 | 6 | 3/8 | 3/8 | 3 Sp, 2 LWR, 1 Diet | 3 Sp, 2 LWR, 1 Diet |
| *Symphurus nigrescens* | Tonguesole | Cynoglossidae | LC | VH | **9** | 6 | 3/8 | 3/8 | **5 LWR**, **2 Sp**, 2 Diet | 3 LWR, 2 Diet, 1 Sp |
| *Argyrosomus regius* | Meagre | Sciaenidae | LC | M | **9** | 5 | **6/8** | 3/8 | 3 Sp, **2 LWR**, 1 G, **1 A**, 1 Mat, **1 Diet** | 3 Spawn, 1 G, 1 Mat |
| *Anthias anthias* | Swallowtail seaperch | Serranidae | LC | VH | **4** | 3 | 3/8 | 3/8 | **2 Diet**, 1 LWR, 1 Sp | 1 Diet, 1 LWR, 1 Sp |
| *Bathysolea profundicola* | Deepwater sole | Soleidae | LC | VH | 3 | 3 | 3/8 | 3/8 | 1 G, 1 Sp, 1 Mat | 1 G, 1 Sp, 1 Mat |
| *Galeorhinus galeus* | Tope shark | Triakidae | CR | M | **4** | 3 | **4/8** | 3/8 | 1 G, 1 Mat, 1 Fec, **1 Diet** | 1 G, 1 Mat, 1 Fec |
| *Microchirus ocellatus* | Foureyed sole | Soleidae | DD | VH | 3 | 3 | 2/8 | 2/8 | 2 LWR, 1 Sp | 2 LWR, 1 Sp |
| *Lepidotrigla dieuzeidei* | Spiny gurnard | Triglidae | LC | VH | **10** | 1 | **6/8** | 1/8 | **3 LWR**, **2 G**, **2 A**, **1 Sp**, **1 Mat**, **1 Fec** | 1 LWR |
| *Thalassoma pavo* | Ornate wrasse | Labridae | LC | VH | **2** | 1 | **2/8** | 1/8 | 1 Sp, **1 Diet** | 1 Sp |
| *Centrolophus niger* | Rudderfish | Centrolophidae | LC | VH | 0 | 0 | 0/8 | 0/8 |  |  |
| *Pseudocaranx dentex* | White trevally | Carangidae | LC | VH | 0 | 0 | 0/8 | 0/8 |  |  |
| **Non-commercial species** | | | | | | | | | | |
| *Blennius ocellaris* | Butterfly blenny | Blenniidae | LC | NA | **10** | 8 | **3/8** | 2/8 | **7 LWR**, 2 Sp, **1 Diet** | 6 LWR, 2 Sp |
| *Callionymus lyra* | Dragonet | Callionymidae | LC | NA | 2 | 2 | 2/8 | 2/8 | 1 LWR, 1 Sp | 1 LWR, 1 Sp |
| **Species with atypical life strategies** | | | | | | | | | | |
| *Syngnathus typhle* | Broadnosed pipefish | Syngnathidae | LC | NA | **18** | 14 | 3/8 | 3/8 | **13 LWR**, **3 Diet**, **2 Sp** | 11 LWR, 2 Diet, 1 Sp |
| *Syngnathus abaster* | Black-striped pipefish | Syngnathidae | LC | NA | **16** | 10 | **4/8** | 3/8 | **9 LWR**, **4 Sp**, **2 Diet**, **1 Fec** | 7 LWR, 2 Sp, 1 Diet |
| *Hippocampus hippocampus* | Short snouted seahorse | Syngnathidae | DD | NA | 7 | 7 | 3/8 | 3/8 | 4 LWR, 2 Diet, 1 Sp | 4 LWR, 2 Diet, 1 Sp |
| *Dasyatis marmorata* | Marbled stingray | Dasyatidae | DD | NA | **10** | 3 | **6/8** | 3/8 | **4 LWR**, **2 Mat**, **1 G**, **1 A**, 1 Fec, 1 Diet | 1 Mat, 1 Fec, 1 Diet |
| *Dasyatis tortonesei* | Tortonese's stingray | Dasyatidae | NA | NA | 3 | 3 | 3/8 | 3/8 | 1 LWR, 1 Mat, 1 Fec | 1 LWR, 1 Mat, 1 Fec |
| *Heptranchias perlo* | Sharpnose sevengill shark | Hexanchidae | NT | NA | **8** | 3 | **5/8** | 3/8 | **4 LWR**, **1 Sp**, 1 Mat, 1 Fec, **1 Diet** | 1 LWR, 1 Mat, 1 Fec |
| *Squatina aculeata* | Sawback angelshark | Squatinidae | CR | M | **16** | 3 | **5/8** | 3/8 | **6 LWR**, **3 Sp**, **3 Mat**, **3 Fec**, **1 Diet** | 1 Sp, 1 Mat, 1 Fec |
| *Squatina squatina* | Angelshark | Squatinidae | CR | M | **6** | 3 | **4/8** | 3/8 | **3 LWR**, 1 Sp, 1 Mat, 1 Fec | 1 Sp, 1 Mat, 1 Fec |
| *Gasterosteus aculeatus* | Three-spined stickleback | Gasterosteidae | LC | NA | 2 | 2 | 2/8 | 2/8 | 1 LWR, 1 Sp | 1 LWR, 1 Sp |
| *Leucoraja melitensis* | Maltese ray | Rajidae | CR | NA | **5** | 2 | **3/8** | 2/8 | **3 LWR**, 1 Sp, 1 Fec | 1 Sp, 1 Fec |
| *Squatina oculata* | Smoothback angelshark | Squatinidae | CR | M | **5** | 2 | **4/8** | 2/8 | **2 LWR**, 1 Sp, 1 Fec, **1 Diet** | 1 Sp, 1 Fec |
| *Aetomylaeus bovinus* | Bull ray | Myliobatidae | CR | M | **11** | 1 | **6/8** | 1/8 | **4 LWR**, **2 Sp**, **2 Diet**, **1 G**, **1 A**, 1 Fec | 1 Fec |
| *Alopias superciliosus* | Bigeye thresher | Alopiidae | VU | L | **8** | 0 | **1/8** | 0/8 | **8 LWR** |  |
| *Alopias vulpinus* | Thresher | Alopiidae | VU | H | **2** | 0 | **2/8** | 0/8 | **1 LWR**, **1 Diet** |  |
| *Lampris guttatus* | Opah | Lampridae | LC | VH | 0 | 0 | 0/8 | 0/8 |  |  |
| *Mola mola* | Ocean sunfish | Molidae | VU | NA | 0 | 0 | 0/8 | 0/8 |  |  |
| *Petromyzon marinus* | Sea lamprey | Petromyzontidae | LC | H | 0 | 0 | 0/8 | 0/8 |  |  |
| *Pterois miles* | Devil firefish | Scorpaenidae | LC | NA | **12** | 0 | **7/8** | 0/8 | **3 Diet**, **2 LWR**, **2 Sp**, **2 Mat**,**1 G**, **1 A**, **1 Fec** |  |
| **Protected species** | | | | | | | | | | |
| *Mobula mobular* | Devil fish | Mobulidae | EN | NA | 4 | 0 | **1/8** | 0/8 | **4 LWR** |  |
| *Lamna nasus* | Porbeagle | Lamnidae | VU | M | 3 | 0 | **1/8** | 0/8 | **3 LWR** |  |
| *Isurus oxyrinchus* | Shortfin mako | Lamnidae | NT | M | 1 | 0 | **1/8** | 0/8 | **1 LWR** |  |
| *Cetorhinus maximus* | Basking shark | Cetorhinidae | EN | L | 1 | 0 | **1/8** | 0/8 | **1 LWR** |  |
| *Carcharodon carcharias* | Great white shark | Lamnidae | VU | L | 5 | 0 | **2/8** | 0/8 | **3 Diet**, **2 LWR** |  |
| *Prionace glauca* | Blue shark | Carcharhinidae | NT | M | 6 | 5 | **4/8** | 3/8 | 2 G, 2 A, **1 LWR,** 1 Sp | 2 G, 2 A, 1 Sp |
